# Omnipresent intercorrelations of metabolic syndrome markers in the general population

**DOI:** 10.1371/journal.pone.0328577

**Published:** 2025-08-14

**Authors:** Marina Sanchez Rico, Emmanuel Wiernik, Sofiane Kab, Adeline Renuy, Nicolas Hoertel, Joël Ménard, Marcel Goldberg, Marie Zins, Pierre Meneton

**Affiliations:** 1 AP-HP, DMU Psychiatrie et Addictologie, Hôpital Corentin-Celton, Issy-les-Moulineaux, France; 2 Université Paris Cité, Université Paris-Saclay, Université de Versailles Saint-Quentin-en-Yvelines, INSERM UMS_011, Villejuif, France; 3 Faculté de Médecine, Université Paris Cité, Paris, France; 4 INSERM UMR_1266, Paris, France; 5 INSERM UMR_1142, Sorbonne Université, Université Paris 13, Paris, France; Universidade Federal de Minas Gerais, BRAZIL

## Abstract

**Background:**

Besides the usual characterization of metabolic syndrome as a cluster of markers arbitrarily defined by thresholds, it is unclear to which extent these markers as continuous traits are correlated with each other in the general population. The present study aimed to explore these correlations across a wide array of biological, social and behavioral characteristics.

**Methods:**

The cross-sectional analyses were performed in a large population-based French cohort (CONSTANCES) of 159,476 adults in whom blood glucose, low-density lipoproteins (LDL) and high-density lipoproteins (HDL), triglycerides, body mass index, waist and hip circumferences, systolic and diastolic blood pressures were measured at the time of recruitment between 2012 and 2021. Correlations between each pair of continuous marker distributions were assessed by calculating raw and partial correlation coefficients (r).

**Results:**

The same pattern of partial correlations is observed with little variation in all groups of sex, age, individual and parental histories of cardiovascular disease, diagnosis of metabolic syndrome, social position, work environment, lifetime unemployment exposure, smoking, non-moderate alcohol consumption, leisure-time physical inactivity and diet quality. This pattern is composed of strong and expected intercorrelations between systolic and diastolic blood pressures (r ranging from 0.62 to 0.74), between body mass index and waist (r from 0.50 to 0.63) and hip (r from 0.58 to 0.70) circumferences and between waist and hip circumferences (r from 0.07 to 0.19). It also includes intercorrelations of systolic blood pressure with waist (r from 0.10 to 0.21) and hip (r from −0.07 to −0.12) circumferences and with blood glucose (r from 0.09 to 0.15), those of triglycerides with blood glucose (r from 0.07 to 0.16), LDL (r from 0.24 to 0.33), HDL (r from −0.20 to −0.29) and waist circumference (r from 0.07 to 0.15), and finally those of waist and hip circumferences with blood glucose (r from 0.09 to 0.17 and from −0.08 to −0.13) and HDL (r from −0.12 to −0.24 and from 0.08 to 0.18).

**Conclusions:**

These results show that metabolic syndrome markers are correlated with each other whatever the biological, social or behavioral characteristics of individuals. They suggest that it makes sense to systematically consider these markers all together rather than separately in terms of etiology, prevention and treatment of metabolic diseases and cardiovascular risk in the general population.

## Introduction

Metabolic syndrome is defined as a cluster of pathological conditions that typically includes hyperglycemia, hypercholesterolemia, hypertriglyceridemia, low HDL cholesterol, high body mass index, abdominal obesity and high blood pressure among several other conditions such as insulin resistance, endothelial dysfunction, hypercoagulable state and vascular inflammation [1]. As such, metabolic syndrome is a major determinant of the risk of cardiovascular diseases [2], including coronary heart disease [3], stroke [4] and probably peripheral arterial disease [5].

A large body of literature shows that many biological, environmental and socioeconomic factors influence the prevalence and incidence of metabolic syndrome. For example, significant sex differences exist in propensity to develop metabolic syndrome and on its impact on cardiovascular risk [6,7]. Metabolic syndrome is obviously more frequent in old than in young individuals [8,9]. It is also associated with low socio-economic indicators [10,11] and poor working conditions [12,13]. Likewise, several behaviors such as smoking [14], heavy alcohol consumption [15], leisure-time physical inactivity [16] and unbalanced diet [17] are associated with metabolic syndrome.

Most studies report the prevalence or incidence of metabolic syndrome by analyzing the co-occurrence of markers which are defined by thresholds, more rarely by measuring the correlations between their continuous distributions. The present study aims to systematically assess these intercorrelations across a wide array of biological, social and behavioral characteristics of individuals in a large population-based cohort. The ubiquity and strength of these intercorrelations may give information to which extent metabolic syndrome markers should be considered all together rather than separately in terms of etiology, prevention and treatment of metabolic diseases and cardiovascular risk in the general population.

## Methods

### Study design

The study retrospectively explored cross-sectional correlations between metabolic syndrome markers across a large array of biological, social and behavioral characteristics of individuals.

### Ethical consideration

The study received approval on September 7, 2021 from both the Ethics Evaluation Committee of the French National Institute of Health and Medical Research (Opinion number 21–842) and the National Committee for the Protection of Privacy and Civil Liberties (Authorization #910486). The authors had no access to information that could identify individual participants during or after data collection.

### Study setting

The analyses were performed in the population-based CONSTANCES cohort whose participants were recruited between February 1, 2012 and September 30, 2021 across French metropolitan territory [18]. These participants who were affiliated to the general health insurance system (which covers 85% of the French population) were selected using a random sampling scheme stratified on sex, age, socioeconomic status and region. Inclusion criteria comprised the obligation to provide written informed consent, to undergo a comprehensive health examination in one of the twenty-one participating medical centers scattered across the territory and to complete questionnaires on lifestyle, health-related behaviors, social and occupational conditions.

### Participants

**Fig 1:**
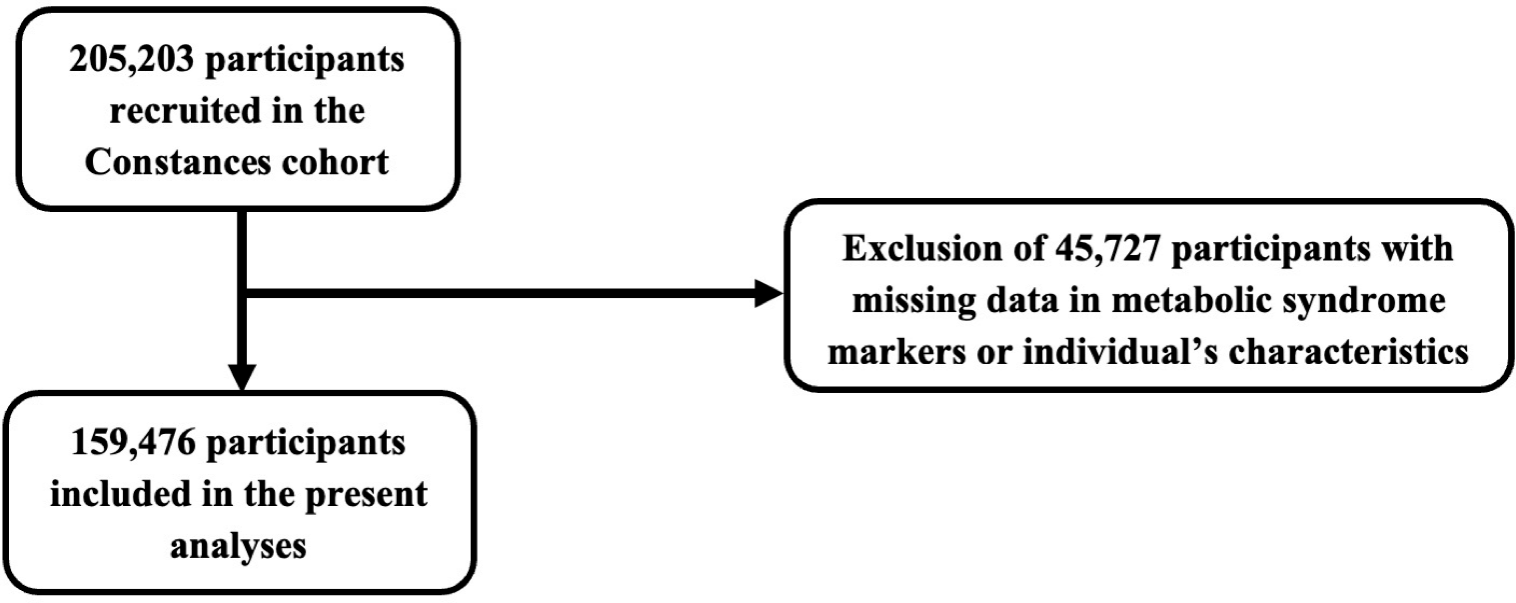
Study flow chart.

From the 205,203 participants who were originally recruited in the cohort, 45,727 were excluded due to missing data in metabolic syndrome markers or individual’s characteristics, leaving 159,476 participants in whom the present analyses were performed (Fig 1).

### Variables

The study investigated nine metabolic syndrome markers, namely glucose, low-density lipoprotein (LDL), high-density lipoprotein (HDL) and triglyceride blood levels, waist and hip circumferences, body mass index, systolic and diastolic blood pressures. The analyses were stratified across a wide array of biological, social and behavioral characteristics of participants including sex, age, individual and parental histories of cardiovascular disease, diagnosis of metabolic syndrome, smoking, non-moderate alcohol consumption, leisure-time physical inactivity, unbalanced diet, social position, work environment and lifetime unemployment exposure.

### Data sources/measurement

Metabolic syndrome markers were assessed at the time of recruitment in the cohort during the visit in the medical centers using standardized procedures in order to guarantee high-quality data [19]. Participants were instructed to fast before the visit that took place between 8 and 10 am, ensuring a fasting period of at least 8h. Waist and hip circumferences were measured as well as height and weight for the calculation of body mass index. Blood was collected to measure concentrations of glucose, total cholesterol, HDL and triglycerides (LDL concentration was calculated by the formula total cholesterol minus HDL minus triglycerides/5) using an analyzer Architect c8000 from Abbott Diagnostics. Systolic and diastolic blood pressures were measured twice in sitting position on each arm at two-minute interval after five minutes of rest using an automated sphygmomanometer OMRON 705. The arm giving the highest mean of the two measures was considered as the reference arm on which a third measure was taken after one additional minute of rest, the average of these three measures was retained for the analyses. The diagnosis of metabolic syndrome was classically defined by the presence of any three of the following five criteria: high waist circumference (≥102 in men or ≥88 cm in women), high blood triglycerides (≥1.7 mmol/l), low blood HDL (<1.0 in men or <1.3 mmol/l in women), high blood pressure (≥130 or ≥85 mmHg for systolic and diastolic blood pressure), high blood glucose (≥5.5 mmol/l) [20].

The other characteristics of individuals, across which intercorrelations of metabolic syndrome markers were tested, were also assessed at the time of recruitment. Besides sex and age, individual history of cardiovascular disease was investigated during the visit in the medical centers where physicians inquired about any non-fatal cardiovascular disease that occurred during the lifetime of participants; four types of cardiovascular diseases were retained for the analyses: stroke, angina pectoris, myocardial infarction and peripheral arterial disease. Parental history of cardiovascular disease referred to the occurrence of at least one of the four cardiovascular diseases on father’s or mother’s side, whatever their age.

Information on risky behaviors (smoking, non-moderate alcohol consumption, leisure-time physical inactivity, unbalanced diet) and socioeconomic conditions (social position, work environment, unemployment exposure) was collected through detailed questionnaires during the same visit. The inquiry assessed habitual smoking status (current, former, never) and the frequency of non-moderate alcohol consumption (more than two or three drinks on the same day in women or men, respectively) classified as rarely (never or less than one time per month), sometimes (two or three times per month) or often (one time or more per week). The assessment of leisure-time physical inactivity was based on a three item questionnaire asking about regular practice of walking or cycling, practicing a sport and gardening or housekeeping over the past 12 months; each item was noted 0 if the answer was no, 1 if the practice was regular but low (less than 15 minutes for sport, or 2 hours for the two other items, per week), 2 if the practice was regular and higher; the score calculated by summing the three items ranged from 0 (not active at all) to 6 (very active) and was used to characterize leisure-time physical inactivity (participants with a score <2). Diet quality was assessed through a weekly 32-item food frequency questionnaire. Each item represented the daily frequency of consumption of a food category on a four-level scale; a score of 0 was given to the frequency “never or almost never”, 2 to “once or twice”, 4 to “not every day but more than twice” and 7 to “every day or almost every day”. The mean of the scores was calculated separately for “healthy items” (poultry, fish, wholewheat bread, whole grain pasta, brown rice, fresh fruits, vegetables, legumes) and “unhealthy items” (red meat, cooked pork meats, white bread, delicacies, appetizers, prepared foods, fast foods, fried foods, pastries, sweet desserts). The two means were divided into terciles and the diet quality was defined by combining the low tercile for unhealthy items with the high tercile for healthy items (balanced diet), the two middle terciles (slightly unbalanced diet) and the high tercile for unhealthy foods with the low tercile for healthy foods (strongly unbalanced diet).

Several indicators whose distributions are shown in S1 Table were used to assess social position of participants. Given that these indicators which included educational attainment, occupation of participants and spouses, monthly income of all household members and an evaluation of social vulnerability described complementary and interdependent aspects of social position, a global score combining all the indicators was calculated and categorized into terciles to delineate low, middle or high social position as previously reported [21]. A series of 19 organizational, physical, biomechanical, chemical and psychosocial exposures were used to characterize work environment of participants that was considered as a whole, which is reality for workers who are not facing only one or a few occupational exposures. For that purpose, these exposures whose distributions are shown in S2 Table were combined into a global score which was categorized into terciles to define bad, average or good work environment as previously described [22]. Unemployment exposure of participants during their lifetime was documented by a questionnaire in which they were asked to report each time they were unemployed for a period of more than six months. The existence of past episodes of unemployment was confirmed for each participant by administrative data from the French national pension system which also provided the total number of unemployed quarters. This number whose distribution is reported in S1 Fig was arbitrarily categorized into three groups (0, 1–19, 20–148 quarters) to estimate unemployment exposure [23].

### Bias

The choice of selecting participants with no missing data rather than imputing them was driven by the fact that the cohort was not representative of the French population. This was due to a low inclusion rate (7.3%) despite the stratified sampling strategy that tried to compensate for the higher non-response rate of individuals with low socioeconomic status [24]. Although in line with rates observed in other large population-based cohorts when participants are required to visit a medical center for health-related exams [25], this low rate resulted in the selection of socially privileged people (S3 Table). The selection of participants with no missing values only marginally accentuated this bias while the alternative of using multivariate imputation by chained equations would not have been devoid of other biases [26]. The means and standard deviations of metabolic syndrome marker distributions were also only marginally affected by the exclusion of participants with missing values as reported in S4 Table.

### Study size

Exact calculation using a series expansion for the distributions of multiple correlation coefficients [27] showed that the minimum sample size to detect with a high power (99%) very significant (p < 0.0001) partial correlations with coefficients of at least 0.1 between 9 continuous variables was n = 5636. Thus, the present study with a population size 28 times larger than required (n = 159,476) was highly powered to reject null hypotheses and explore partial correlations between metabolic syndrome markers.

### Quantitative variables

Metabolic syndrome markers (blood glucose, low-density and high-density lipoproteins, triglycerides, body mass index, waist and hip circumferences, systolic and diastolic blood pressures) were analyzed as continuous variables to test their intercorrelations. Sex (F/M), individual and parental histories of cardiovascular disease (N/Y), diagnosis of metabolic syndrome (N/Y) and leisure-time physical inactivity (N/Y) were coded as binary variables. Age was divided into terciles while smoking (never, former, current), non-moderate alcohol consumption (rarely, sometimes, often), unbalanced diet (no, slightly, strongly), social position (high, middle, low), work environment (good, average, bad) and lifetime unemployment exposure (0, 1–19, 20–148 quarters) were coded into three categories as mentioned above.

### Statistical methods

The strength of the correlations between metabolic syndrome markers were measured by calculating Pearson correlation coefficients between each pair of markers as well as partial correlation coefficients that measured the strength of the correlations after adjusting for the effects of all the other markers. The analyses were stratified across a wide array of biological, social and behavioral characteristics of participants that included sex, age, individual and parental histories of cardiovascular disease, diagnosis of metabolic syndrome, smoking, non-moderate alcohol consumption, leisure-time physical inactivity, unbalanced diet, social position, work environment and lifetime unemployment exposure. Given that partial correlations between each pair of metabolic syndrome markers (n = 36) were assessed 32 times across biological, social and behavioral characteristics of participants, the statistical significance was set at p < 0.0001 to minimize as much as possible the risk of having false positives due to multiple testing. All analyses were performed with statistical discovery software JMP 17 Pro (SAS, Cary NC).

## Results

### Characteristics of participants

The characteristics of the 159,476 participants selected for this study are reported in Table 1. Aged in average 46.5 years (SD 13.4), 2.1 and 25.0% of them had respectively an individual or a parental history of cardiovascular disease. While a large fraction (62.1%) often consumed alcohol in a non-moderate way and 53.6% were current (18.7%) or former (34.8%) smokers, only 9.3% were physically inactive during their leisure-time and 10.4% had a strongly unbalanced diet. A significant proportion had a low social position (25.7%) or were exposed to bad work environment (32.5%) but only 14.8% were exposed to short (9.8%) or long (5.0%) periods of unemployment during their lifetime.

**Table 1:** Characteristics of participants.

		n	%
**Sex**	**Women**	75,210	47.2
	**Men**	84,266	52.8
**Age**	**18-39**	50,782	31.8
	**40-55**	54,496	34.2
	**56-75**	54,198	34.0
**Individual history of** **cardiovascular disease**	**No**	156,147	97.9
	**Yes**	3329	2.1
**Parental history of** **cardiovascular disease**	**No**	119,538	75.0
	**Yes**	39,938	25.0
**Non-moderate** **alcohol consumption**	**Rarely**	28,172	17.7
	**Sometimes**	32,291	20.2
	**Often**	99,013	62.1
**Smoking**	**Never**	74,095	46.5
	**Former**	55,594	34.8
	**Current**	29,787	18.7
**Leisure-time** **physical inactivity**	**No**	144,637	90.7
	**Yes**	14,839	9.3
**Unbalanced diet**	**No**	19,304	12.1
	**Slightly**	123,518	77.5
	**Strongly**	16,654	10.4
**Social position**	**High**	43,594	27.3
	**Middle**	74,943	47.0
	**Low**	40,939	25.7
**Work environment**	**Good**	52,629	33.0
	**Average**	55,054	34.5
	**Bad**	51,793	32.5
**Lifetime unemployment** **exposure (quarters)**	**0**	135,982	85.2
	**1-19**	15,562	9.8
	**20-148**	7932	5.0

The percentages were calculated relatively to the number of participants selected for the study (n=159,476).

**Fig 2:**
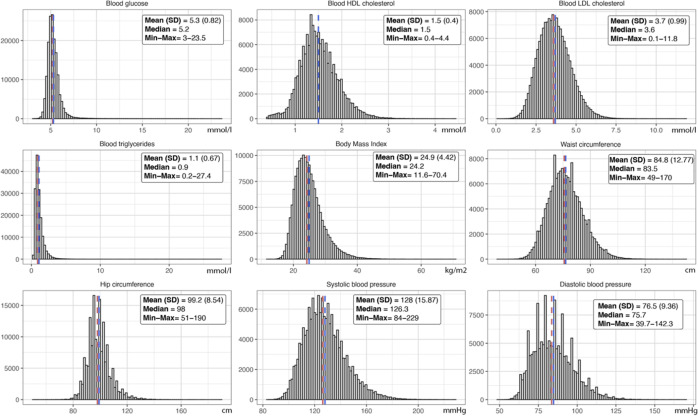
Distributions of metabolic syndrome markers in participants. The mean with standard deviation (SD) and median as well as minimum and maximum values are reported for each marker.

Continuous distributions of metabolic syndrome markers in participants are shown in Fig 2. Average blood concentrations (SD) were respectively 5.31 (0.82), 1.51 (0.40), 3.66 (0.99) and 1.12 (0.67) mmol/l for glucose, HDL cholesterol, LDL cholesterol and triglycerides. Average values (SD) were 24.9 (4.4) kg/m^2^ for body mass index, 84.8 (12.8) and 99.2 (8.5) cm for waist and hip circumferences, 128.0 (15.8) and 76.5 (9.4) mmHg for systolic and diastolic blood pressures. Metabolic syndrome was diagnosed in 13.8% of participants (n = 22,044).

### Correlations and partial correlations between metabolic syndrome markers in all participants

Correlations and partial correlations coefficients between metabolic syndrome markers in all participants are reported in Table 2. Although each marker was significantly correlated with all the others, partial correlations coefficients indicate that some markers were more strongly intercorrelated than others when adjusting for the effects of the other markers. Besides the strong and expected partial correlations between systolic and diastolic blood pressures (r = 0.71), between body mass index and waist (r = 0.55) and hip (r = 0.63) circumferences and between waist and hip circumferences (r = 0.18), systolic blood pressure was correlated with waist (r = 0.17) and hip (r = −0.10) circumferences and with blood glucose (r = 0.13), triglycerides with blood glucose (r = 0.11), LDL (r = 0.27), HDL (r = −0.23) and waist circumference (r = 0.12), waist and hip circumferences with blood glucose (r = 0.16 and −0.11) and HDL (r = −0.16 and 0.10).

**Table 2:** Correlations and partial correlations between metabolic syndrome markers in all participants.

	Blood glucose	Blood LDL cholesterol	Blood HDL cholesterol	Blood triglycerides	Body mass index	Waist circumference	Hip circumference	Systolic blood pressure
**Blood LDL** **cholesterol**	0.1380 −0.0285	.	.	.	.	.	.	.
**Blood HDL** **cholesterol**	−0.1608 0.0024	−0.1241 0.0290	.	.	.	.	.	.
**Blood** **triglycerides**	**0.2756** **0.1113**	**0.3602** **0.2673**	**−0.3486** **−0.2344**	.	.	.	.	.
**Body mass** **index**	0.3227 0.0706	0.2314 0.0517	−0.3009 −0.0652	0.3474 0.0393	.	.	.	.
**Waist** **circumference**	**0.3911** **0.1605**	0.2604 0.0363	**−0.3524** **−0.1616**	**0.4071** **0.1173**	**0.8414** **0.5478**	.	.	.
**Hip** **circumference**	**0.1830** **−0.1144**	0.1324 −0.0539	**−0.1874** **0.0995**	0.2159 −0.0587	**0.8249** **0.6364**	**0.6876** **0.1831**	.	.
**Systolic blood** **pressure**	**0.3194** **0.1328**	0.2493 0.0446	−0.0896 0.0737	0.2508 0.0054	0.3195 −0.0093	**0.4068** **0.1680**	**0.1709** **−0.1004**	.
**Diastolic blood** **pressure**	0.2530 −0.0270	0.2546 0.0673	−0.1046 −0.0053	0.2508 0.0379	0.3319 0.0657	0.3781 −0.0169	0.2033 0.0010	**0.7664** **0.7089**

For each pair of markers, the top-line shows correlation coefficients while the bottom-line reports partial correlation coefficients. Note that correlation and partial correlation coefficients can have different signs because partial correlations measure the strength of the correlations between each pair of markers after adjusting for the effects of all the other markers. Gray background indicates significant partial correlations with p<0.0001 while bold characters underline the strongest partial correlation coefficients r ≥ 0.1.

### Partial correlations between metabolic syndrome according to sex

The same pattern of partial correlations described in all participants was observed with little differences in both sexes (Table 3). In women and men, there were strong correlations between systolic and diastolic blood pressures (r = 0.72 and 0.69), between body mass index and waist (r = 0.52 and 0.63) and hip (r = 0.61 and 0.59) circumferences and between waist and hip circumferences (r = 0.18 and 0.19); systolic blood pressure was correlated with waist (r = 0.16 and 0.16) and hip (r = −0.09 and −0.11) circumferences and with blood glucose (r = 0.14 and 0.11), triglycerides with blood glucose (r = 0.12 and 0.11), LDL (r = 0.30 and 0.25), HDL (r = −0.20 and −0.29) and waist circumference (r = 0.14 and 0.09), HDL also with waist (r = −0.20 and −0.23) and hip (r = 0.14 and 0.11) circumferences, blood glucose with waist (r = 0.10 and 0.15) and hip (r = −0.08 and −0.13) circumferences.

**Table 3:** Partial correlations between metabolic syndrome markers according to sex.

	Blood glucose	Blood LDL cholesterol	Blood HDL cholesterol	Blood triglycerides	Body mass index	Waist circumference	Hip circumference	Systolic blood pressure
**Blood LDL** **cholesterol**	0.0106	.	.	.	.	.	.	.
	−0.0569	.	.	.	.	.	.	.
**Blood HDL** **cholesterol**	0.0087	0.0154	.	.	.	.	.	.
	0.0071	0.0044	.	.	.	.	.	.
**Blood** **triglycerides**	**0.1156**	**0.3027**	**−0.2037**	.	.	.	.	.
	**0.1069**	**0.2463**	**−0.2899**	.	.	.	.	.
**Body mass** **index**	0.0818	0.0431	−0.1271	0.0500	.	.	.	.
	0.0543	0.0171	−0.0829	0.0300	.	.	.	.
**Waist** **circumference**	**0.0999**	0.0711	**−0.2006**	**0.1386**	**0.5191**	.	.	.
	**0.1517**	0.0584	**−0.2258**	**0.0872**	**0.6347**	.	.	.
**Hip** **circumference**	**−0.0786**	−0.0863	**0.1386**	−0.0806	**0.6116**	**0.1820**	.	.
	**−0.1268**	−0.0504	**0.1092**	−0.0466	**0.5895**	**0.1936**	.	.
**Systolic blood** **pressure**	**0.1407**	0.1163	0.1369	0.0454	0.0154	**0.1649**	**−0.0946**	.
	**0.1136**	−0.0354	0.0800	−0.0029	0.0255	**0.1556**	**−0.1059**	.
**Diastolic blood** **pressure**	−0.0361	0.0028	−0.0417	0.0101	0.0648	−0.0236	0.0101	**0.7247**
	−0.0207	0.0142	0.0051	0.0451	0.0288	0.0680	−0.0515	**0.6951**

For each pair of markers, the top and bottom-lines show respectively partial correlation coefficients in women and men. Gray background indicates significant partial correlations with p<0.0001 while bold characters underline the strongest partial correlation coefficients r ≥ 0.1.

### Partial correlations between metabolic syndrome markers according to age

The same pattern of partial correlations was observed whatever the age group (Table 4). In 18−39, 40−55 and 56−75 years-old participants, there were strong correlations between systolic and diastolic blood pressures (r = 0.64, 0.69 and 0.71), between body mass index, waist (r = 0.50, 0.53 and 0.55) and hip (r = 0.61, 0.63 and 0.65) circumferences and between waist and hip circumferences (r = 0.17, 0.11 and 0.17); systolic blood pressure was correlated with waist (r = 0.16, 0.15 and 0.14) and hip (r = −0.11, −0.07 and −0.10) circumferences and with blood glucose (r = 0.13, 0.09 and 0.11), triglycerides with blood glucose (r = 0.13, 0.10 and 0.15), LDL (r = 0.28, 0.26 and 0.25), HDL (r = −0.26, −0.23 and −0.26) and waist circumference (r = 0.13, 0.14 and 0.08), HDL also with waist (r = −0.12, −0.18 and −0.24) and hip (r = 0.09, 0.12 and 0.17) circumferences, blood glucose with waist (r = 0.09, 0,13 and 0.14) and hip (r = −0.08, −0.09 and −0.09) circumferences.

**Table 4:** Partial correlations between metabolic syndrome markers according to age.

	Blood glucose	Blood LDL cholesterol	Blood HDL cholesterol	Blood triglycerides	Body mass index	Waist circumference	Hip circumference	Systolic blood pressure
**Blood LDL** **cholesterol**	0.0361	.	.	.	.	.	.	.
	−0.0103	.	.	.	.	.	.	.
	−0.1118	.	.	.	.	.	.	.
**Blood HDL** **cholesterol**	−0.0396	−0.0609	.	.	.	.	.	.
	−0.0162	−0.0607	.	.	.	.	.	.
	−0.0284	−0.0355	.	.	.	.	.	.
**Blood** **triglycerides**	**0.1323**	**0.2845**	**−0.2627**	.	.	.	.	.
	**0.1038**	**0.2556**	**−0.2263**	.	.	.	.	.
	**0.1550**	**0.2485**	**−0.2564**	.	.	.	.	.
**Body mass** **index**	0.0281	0.0556	−0.0788	0.0248	.	.	.	.
	0.0694	0.0355	−0.0795	0.0298	.	.	.	.
	0.0751	0.0263	−0.0681	0.0610	.	.	.	.
**Waist** **circumference**	**0.0937**	0.0813	**−0.1228**	**0.1281**	**0.4989**	.	.	.
	**0.1267**	0.0517	**−0.1782**	**0.1408**	**0.5289**	.	.	.
	**0.1374**	−0.1143	**−0.2445**	**0.0782**	**0.5554**	.	.	.
**Hip** **circumference**	**−0.0825**	−0.0517	**0.0934**	−0.0571	**0.6144**	**0.1711**	.	.
	**−0.0912**	−0.0563	**0.1211**	−0.0744	**0.6342**	**0.1087**	.	.
	**−0.0934**	0.0373	**0.1660**	−0.0448	**0.6470**	**0.1684**	.	.
**Systolic blood** **pressure**	**0.1344**	−0.0189	−0.0509	0.0054	−0.0488	**0.1620**	**−0.1060**	.
	**0.0924**	0.0349	0.0502	0.0006	−0.0238	**0.1550**	**−0.0719**	.
	**0.1062**	−0.0098	0.0512	0.0234	0.0177	**0.1436**	**−0.0976**	.
**Diastolic blood** **pressure**	−0.0026	0.0874	0.0586	0.0605	0.0968	−0.1174	0.0335	**0.6398**
	−0.0184	0.0314	−0.0137	0.0462	0.0759	−0.0424	0.0027	**0.6880**
	−0.0427	0.0559	−0.0303	0.0080	0.0262	0.0634	−0.0188	**0.7059**

For each pair of markers, the top, middle and bottom-lines show respectively partial correlation coefficients for 18-39, 40-55 and 56-75 years-old participants. Gray background indicates significant partial correlations with p<0.0001 while bold characters underline the strongest partial correlation coefficients r ≥ 0.1.

### Partial correlations between metabolic syndrome markers according to individual and parental history of cardiovascular disease

Neither individual or parental histories of cardiovascular disease substantially modified the pattern of partial correlations between metabolic syndrome markers. In participants without or with individual history of cardiovascular disease (Table 5), there were strong correlations between systolic and diastolic blood pressures (r = 0.71 and 0.66), between body mass index, and waist (r = 0.54 and 0.63) and hip (r = 0.64 and 0.63) circumferences and between waist and hip circumferences (r = 0.09 and 0.11); systolic blood pressure was correlated with waist (r = 0.16 and 0.12) and hip (r = −0.10 and −0.09) circumferences and with blood glucose (r = 0.13 and 0.13), triglycerides with blood glucose (r = 0.11 and 0.08), LDL (r = 0.27 and 0.27), HDL (r = −0.24 and −0.24) and waist circumference (r = 0.11 and 0.15), HDL also with waist (r = −0.17 and −0.21) and hip (r = 0.10 and 0.18) circumferences, blood glucose with waist (r = 0.16 and 0.12) and hip (r = −0.11 and −0.11) circumferences. In participants without or with parental history of cardiovascular disease (Table 6), there were strong correlations between systolic and diastolic blood pressures (r = 0.71 and 0.71), between body mass index and waist (r = 0.54 and 0.56) and hip (r = 0.63 and 0.66) circumferences and between waist and hip circumferences (r = 0.09 and 0.15); systolic blood pressure was correlated with waist (r = 0.18 and 0.13) and hip (r = −0.10 and −0.10) circumferences and with blood glucose (r = 0.13 and 0.14), triglycerides with blood glucose (r = 0.10 and 0.13), LDL (r = 0.27 and 0.26), HDL (r = −0.23 and −0.24) and waist circumference (r = 0.12 and 0.11), HDL also with waist (r = −0.15 and −0.19) and hip (r = 0.09 and 0.12) circumferences, blood glucose with waist (r = 0.16 and 0.15) and hip (r = −0.11 and −0.11) circumferences.

**Table 5:** Partial correlations between metabolic syndrome markers according to individual history of cardiovascular disease.

	Blood glucose	Blood LDL cholesterol	Blood HDL cholesterol	Blood triglycerides	Body mass index	Waist circumference	Hip circumference	Systolic blood pressure
**Blood LDL** **cholesterol**	−0.0158	.	.	.	.	.	.	.
	−0.0904	.	.	.	.	.	.	.
**Blood HDL** **cholesterol**	0.0073	0.0356	.	.	.	.	.	.
	−0.0363	0.0953	.	.	.	.	.	.
**Blood** **triglycerides**	**0.1055**	**0.2717**	**−0.2393**	.	.	.	.	.
	**0.0826**	**0.2666**	**−0.2434**	.	.	.	.	.
**Body mass** **index**	0.0682	0.0520	−0.0600	0.0406	.	.	.	.
	0.0858	0.0177	−0.0741	0.0679	.	.	.	.
**Waist** **circumference**	**0.1570**	0.0512	**−0.1667**	**0.1130**	**0.5420**	.	.	.
	**0.1207**	−0.0999	**−0.2111**	**0.1497**	**0.6346**	.	.	.
**Hip** **circumference**	**−0.1094**	−0.0625	**0.0959**	−0.0575	**0.6366**	**0.0893**	.	.
	**−0.1154**	0.0542	**0.1784**	−0.0679	**0.6331**	**0.1136**	.	.
**Systolic blood** **pressure**	**0.1304**	0.0526	0.0706	0.0022	−0.0092	**0.1636**	**−0.0973**	.
	**0.1280**	−0.0057	0.0535	0.0188	−0.0220	**0.1220**	**−0.0864**	.
**Diastolic blood** **pressure**	−0.0223	0.0576	−0.0026	0.0413	0.0669	−0.0141	−0.0020	**0.7110**
	−0.0792	0.0942	0.0014	0.0158	0.0416	−0.0005	0.0108	**0.6648**

For each pair of markers, the top and bottom-lines show respectively partial correlation coefficients in participants without or with individual history of cardiovascular disease. Gray background indicates significant partial correlations with p<0.0001 while bold characters underline the strongest partial correlation coefficients r ≥ 0.1.

**Table 6:** Partial correlations between metabolic syndrome markers according to parental history of cardiovascular disease.

	Blood glucose	Blood LDL cholesterol	Blood HDL cholesterol	Blood triglycerides	Body mass index	Waist circumference	Hip circumference	Systolic blood pressure
**Blood LDL** **cholesterol**	−0.0150	.	.	.	.	.	.	.
	−0.0636	.	.	.	.	.	.	.
**Blood HDL** **cholesterol**	0.0031	0.0243	.	.	.	.	.	.
	−0.0042	0.0165	.	.	.	.	.	.
**Blood** **triglycerides**	**0.1017**	**0.2690**	**−0.2295**	.	.	.	.	.
	**0.1328**	**0.2604**	**−0.2453**	.	.	.	.	.
**Body mass** **index**	0.0658	0.0544	−0.0658	0.0343	.	.	.	.
	0.0803	0.0375	−0.0685	0.0533	.	.	.	.
**Waist** **circumference**	**0.1630**	0.0502	**−0.1533**	**0.1211**	**0.5428**	.	.	.
	**0.1491**	−0.0121	**−0.1880**	**0.1055**	**0.5570**	.	.	.
**Hip** **circumference**	**−0.1143**	−0.0604	**0.0942**	−0.0567	**0.6288**	**0.0950**	.	.
	**−0.1113**	−0.0266	**0.1203**	−0.0645	**0.6589**	**0.1534**	.	.
**Systolic blood** **pressure**	**0.1298**	0.0367	0.0610	0.0024	−0.0149	**0.1824**	**−0.1018**	.
	**0.1383**	0.0511	0.0967	0.0139	0.0046	**0.1291**	**−0.0965**	.
**Diastolic blood** **pressure**	−0.0187	0.0746	0.0051	0.0409	0.0712	−0.0256	0.0010	**0.7072**
	−0.0487	0.0410	−0.0364	0.0300	0.0489	0.0001	0.0058	**0.7122**

For each pair of markers, the top and bottom-lines show respectively partial correlation coefficients in participants without or with parental history of cardiovascular disease. Gray background indicates significant partial correlations with p<0.0001 while bold characters underline the strongest partial correlation coefficients r ≥ 0.1.

### Partial correlations between metabolic syndrome markers according to the diagnosis of metabolic syndrome

The same pattern of partial correlations was observed independently of the diagnosis of metabolic syndrome (Table 7). In participants without or with metabolic syndrome, there were strong correlations between systolic and diastolic blood pressures (r = 0.72 and 0.62), between body mass index and waist (r = 0.53 and 0.54) and hip (r = 0.61 and 0.70) circumferences and between waist and hip circumferences (r = 0.10 and 0.10); systolic blood pressure was correlated with waist (r = 0.17 and 0.10) and hip (r = −0.10 and −0.11) circumferences and with blood glucose (r = 0.13 and 0.10), triglycerides with blood glucose (r = 0.10 and 0.09), LDL (r = 0.33 and 0.30), HDL (r = −0.22 and −0.22) and waist circumference (r = 0.11 and 0.07), HDL also with waist (r = −0.16 and −0.13) and hip (r = 0.09 and 0.16) circumferences, blood glucose with waist (r = 0.16 and 0.14) and hip (r = −0.11 and −0.13) circumferences.

**Table 7:** Partial correlations between metabolic syndrome markers according to the diagnosis of metabolic syndrome.

	Blood glucose	Blood LDL cholesterol	Blood HDL cholesterol	Blood triglycerides	Body mass index	Waist circumference	Hip circumference	Systolic blood pressure
**Blood LDL** **cholesterol**	0.0426	.	.	.	.	.	.	.
	−0.0125	.	.	.	.	.	.	.
**Blood HDL** **cholesterol**	0.0112	0.0596	.	.	.	.	.	.
	0.0359	0.0639	.	.	.	.	.	.
**Blood** **triglycerides**	**0.1036**	**0.3317**	**−0.2194**	.	.	.	.	.
	**0.0917**	**0.2990**	**−0.2210**	.	.	.	.	.
**Body mass** **index**	0.0345	0.0650	−0.0499	0.0309	.	.	.	.
	0.0678	0.0192	−0.0975	−0.0135	.	.	.	.
**Waist** **circumference**	**0.1565**	0.0453	**−0.1643**	**0.1151**	**0.5305**	.	.	.
	**0.1403**	−0.0887	**−0.1350**	**0.0697**	**0.5443**	.	.	.
**Hip** **circumference**	**−0.1076**	−0.0585	**0.0889**	−0.0594	**0.6125**	**0.0999**	.	.
	**−0.1332**	−0.0175	**0.1582**	−0.0607	**0.7003**	**0.0996**	.	.
**Systolic blood** **pressure**	**0.1333**	0.0405	0.0632	−0.0077	−0.0133	**0.1657**	**−0.1005**	.
	**0.0974**	−0.0199	0.0637	−0.0297	−0.0071	**0.0958**	**−0.1090**	.
**Diastolic blood** **pressure**	−0.0288	0.0580	0.0134	0.0340	0.0669	−0.0284	0.0034	**0.7188**
	−0.0423	0.0863	−0.0393	0.0177	0.0451	0.0010	−0.0005	**0.6186**

For each pair of markers, the top and bottom-lines show respectively partial correlation coefficients in participants without or with metabolic syndrome. Gray background indicates significant partial correlations with p<0.0001 while bold characters underline the strongest partial correlation coefficients r ≥ 0.1.

### Partial correlations between metabolic syndrome markers according to social position

The same pattern of partial correlations was observed across social position (Table 8). In participants with high, middle or low social position, there were strong correlations between systolic and diastolic blood pressures (r = 0.74, 0.71 and 0.69), between body mass index and waist (r = 0.51, 0.54 and 0.57) and hip (r = 0.58, 0.64 and 0.68) circumferences and between waist and hip circumferences (r = 0.15, 0.08 and 0.12); systolic blood pressure was correlated with waist (r = 0.18, 0.18 and 0.15) and hip (r = −0.09, −0.10 and −0.08) circumferences and with blood glucose (r = 0.12, 0.14 and 0.13), triglycerides with blood glucose (r = 0.07, 0.10 and 0.13), LDL (r = 0.30, 0.27 and 0.25), HDL (r = −0.24, −0.22 and −0.25) and waist circumference (r = 0.12, 0.12 and 0.11), HDL also with waist (r = −0.18, −0.15 and −0.15) and hip (r = 0.08, 0.10 and 0.11) circumferences, blood glucose with waist (r = 0.16, 0,16 and 0.16) and hip (r = −0.10, −0.11 and −0.12) circumferences.

**Table 8:** Partial correlations between metabolic syndrome markers according to social position.

	Blood glucose	Blood LDL cholesterol	Blood HDL cholesterol	Blood triglycerides	Body mass index	Waist circumference	Hip circumference	Systolic blood pressure
**Blood LDL** **cholesterol**	0.0058	.	.	.	.	.	.	.
	−0.0140	.	.	.	.	.	.	.
	−0.0511	.	.	.	.	.	.	.
**Blood HDL** **cholesterol**	−0.0001	0.0290	.	.	.	.	.	.
	0.0092	0.0243	.	.	.	.	.	.
	−0.0120	0.0464	.	.	.	.	.	.
**Blood** **triglycerides**	**0.0704**	**0.2981**	**−0.2407**	.	.	.	.	.
	**0.1052**	**0.2670**	**−0.2246**	.	.	.	.	.
	**0.1280**	**0.2538**	**−0.2470**	.	.	.	.	.
**Body mass** **index**	0.0646	0.0474	−0.0514	0.0622	.	.	.	.
	0.0625	0.0630	−0.0759	0.0342	.	.	.	.
	0.0699	0.0384	−0.0653	0.0231	.	.	.	.
**Waist** **circumference**	**0.1629**	0.0539	**−0.1839**	**0.1215**	**0.5086**	.	.	.
	**0.1628**	0.0376	**−0.1542**	**0.1234**	**0.5421**	.	.	.
	**0.1590**	0.0176	**−0.1493**	**0.1106**	**0.5750**	.	.	.
**Hip** **circumference**	**−0.1008**	−0.0502	**0.0841**	−0.0665	**0.5814**	**0.1483**	.	.
	**−0.1110**	−0.0673	**0.0991**	−0.0535	**0.6376**	**0.0842**	.	.
	**−0.1167**	−0.0351	**0.1114**	−0.0512	**0.6831**	**0.1230**	.	.
**Systolic blood** **pressure**	**0.1235**	0.0336	0.0436	0.0054	−0.0117	**0.1771**	**−0.0913**	.
	**0.1367**	0.0478	0.0745	0.0036	−0.0186	**0.1755**	**−0.0986**	.
	**0.1295**	0.0364	0.0977	0.0032	−0.0265	**0.1525**	**−0.0788**	.
**Diastolic blood** **pressure**	−0.0176	0.0631	0.0083	0.0370	0.0706	−0.0389	0.0063	**0.7368**
	−0.0264	0.0619	−0.0090	0.0384	0.0760	−0.0315	0.0022	**0.7126**
	−0.0269	0.0801	−0.0088	0.0421	0.0690	0.0132	−0.0223	**0.6886**

For each pair of markers, the top, middle and bottom-lines show respectively partial correlation coefficients for high, middle and low social position. Gray background indicates significant partial correlations with p<0.0001 while bold characters underline the strongest partial correlation coefficients r ≥ 0.1.

### Partial correlations between metabolic syndrome markers according to work environment

Table 9 shows that the same pattern of partial correlations was observed across work environment. In participants with good, average or bad work environment, there were strong correlations between systolic and diastolic blood pressures (r = 0.74, 0.71 and 0.69), between body mass index and waist (r = 0.50, 0.55 and 0.58) and hip (r = 0.61, 0.65 and 0.66) circumferences and between waist and hip circumferences (r = 0.15, 0.08 and 0.14); systolic blood pressure was correlated with waist (r = 0.17, 0.16 and 0.13) and hip (r = −0.08, −0.09 and −0.09) circumferences and with blood glucose (r = 0.12, 0.13 and 0.12), triglycerides with blood glucose (r = 0.09, 0.10 and 0.13), LDL (r = 0.29, 0.27 and 0.25), HDL (r = −0.23, −0.23 and −0.25) and waist circumference (r = 0.14, 0.12 and 0.08), HDL also with waist (r = −0.17, −0.15 and −0.18) and hip (r = 0.08, 0.10 and 0.13) circumferences, blood glucose with waist (r = 0.14, 0,16 and 0.16) and hip (r = −0.09, −0.11 and −0.12) circumferences.

**Table 9:** Partial correlations between metabolic syndrome according to work environment.

	Blood glucose	Blood LDL cholesterol	Blood HDL cholesterol	Blood triglycerides	Body mass index	Waist circumference	Hip circumference	Systolic blood pressure
**Blood LDL** **cholesterol**	0.0136	.	.	.	.	.	.	.
	−0.0134	.	.	.	.	.	.	.
	−0.0735	.	.	.	.	.	.	.
**Blood HDL** **cholesterol**	0.0077	0.0167	.	.	.	.	.	.
	−0.0014	0.0265	.	.	.	.	.	.
	−0.0067	0.0445	.	.	.	.	.	.
**Blood** **triglycerides**	**0.0878**	**0.2944**	**−0.2327**	.	.	.	.	.
	**0.1015**	**0.2669**	**−0.2303**	.	.	.	.	.
	**0.1320**	**0.2465**	**−0.2496**	.	.	.	.	.
**Body mass** **index**	0.0636	0.0607	−0.0549	0.0414	.	.	.	.
	0.0634	0.0549	−0.0740	0.0290	.	.	.	.
	0.0732	0.0281	−0.0735	0.0500	.	.	.	.
**Waist** **circumference**	**0.1420**	0.0513	**−0.1686**	**0.1397**	**0.5001**	.	.	.
	**0.1611**	0.0448	**−0.1489**	**0.1247**	**0.5456**	.	.	.
	**0.1575**	−0.0103	**−0.1795**	**0.0790**	**0.5770**	.	.	.
**Hip** **circumference**	**−0.0898**	−0.0620	**0.0847**	−0.0682	**0.6118**	**0.1456**	.	.
	**−0.1105**	−0.0635	**0.1017**	−0.0549	**0.6456**	**0.0809**	.	.
	**−0.1164**	−0.0142	**0.1313**	−0.0475	**0.6587**	**0.1377**	.	.
**Systolic blood** **pressure**	**0.1226**	0.0244	0.0180	0.0023	−0.0245	**0.1701**	**−0.0785**	.
	**0.1268**	0.0378	0.0749	0.0105	−0.0197	**0.1612**	**−0.0855**	.
	**0.1231**	0.0397	0.1000	−0.0021	−0.0029	**0.1295**	**−0.0903**	.
**Diastolic blood** **pressure**	−0.0138	0.0687	0.0296	0.0405	0.0798	−0.0382	−0.0003	**0.7371**
	−0.0207	0.0742	−0.0052	0.0375	0.0757	−0.0187	−0.0075	**0.7110**
	−0.0315	0.0657	−0.0234	0.0386	0.0493	0.0292	−0.0162	**0.6903**

For each pair of markers, the top, middle and bottom-lines show respectively partial correlation coefficients for good, average and bad work environment. Gray background indicates significant partial correlations with p<0.0001 while bold characters underline the strongest partial correlation coefficients r ≥ 0.1.

### Partial correlations between metabolic syndrome markers according to lifetime unemployment exposure

The same pattern of partial correlations was observed whatever lifetime unemployment exposure (Table 10). In participants exposed to 0, 1–19 or 20–148 unemployed quarters, there were strong correlations between systolic and diastolic blood pressures (r = 0.71, 0.72 and 0.74), between body mass index and waist (r = 0.55, 0.54 and 0.56) and hip (r = 0.63, 0.66 and 0.70) circumferences and between waist and hip circumferences (r = 0.09, 0.08 and 0.11); systolic blood pressure was correlated with waist (r = 0.17, 0.18 and 0.13) and hip (r = −0.10, −0.09 and −0.07) circumferences and with blood glucose (r = 0.13, 0.13 and 0.12), triglycerides with blood glucose (r = 0.11, 0.11 and 0.16), LDL (r = 0.27, 0.26 and 0.27), HDL (r = −0.23, −0.24 and −0.26) and waist circumference (r = 0.11, 0.14 and 0.14), HDL also with waist (r = −0.16, −0.17 and −0.19) and hip (r = 0.10, 0.10 and 0.12) circumferences, blood glucose with waist (r = 0.16, 0,15 and 0.13) and hip (r = −0.11, −0.11 and −0.11) circumferences.

**Table 10:** Partial correlations between metabolic syndrome markers according to lifetime unemployment exposure.

	Blood glucose	Blood LDL cholesterol	Blood HDL cholesterol	Blood triglycerides	Body mass index	Waist circumference	Hip circumference	Systolic blood pressure
**Blood LDL** **cholesterol**	−0.0249	.	.	.	.	.	.	.
	−0.0243	.	.	.	.	.	.	.
	−0.0835	.	.	.	.	.	.	.
**Blood HDL** **cholesterol**	0.0038	0.0309	.	.	.	.	.	.
	−0.0020	0.0140	.	.	.	.	.	.
	−0.0155	0.0061	.	.	.	.	.	.
**Blood** **triglycerides**	**0.1066**	**0.2673**	**−0.2318**	.	.	.	.	.
	**0.1127**	**0.2639**	**−0.2421**	.	.	.	.	.
	**0.1558**	**0.2676**	**−0.2601**	.	.	.	.	.
**Body mass** **index**	0.0714	0.0500	−0.0658	0.0430	.	.	.	.
	0.0573	0.0580	−0.0650	0.0193	.	.	.	.
	0.0830	0.0529	−0.0654	0.0172	.	.	.	.
**Waist** **circumference**	**0.1629**	0.0411	**−0.1596**	**0.1130**	**0.5481**	.	.	.
	**0.1543**	0.0257	**−0.1664**	**0.1406**	**0.5394**	.	.	.
	**0.1308**	−0.0311	**−0.1865**	**0.1393**	**0.5593**	.	.	.
**Hip** **circumference**	**−0.1149**	−0.0543	**0.0980**	−0.0582	**0.6284**	**0.0881**	.	.
	**−0.1076**	−0.0600	**0.1049**	−0.0558	**0.6597**	**0.0806**	.	.
	**−0.1154**	−0.0205	**0.1224**	−0.0648	**0.7047**	**0.1152**	.	.
**Systolic blood** **pressure**	**0.1346**	0.0434	0.0726	0.0064	−0.0063	**0.1685**	**−0.1025**	.
	**0.1316**	0.0575	0.0714	0.0017	−0.0253	**0.1764**	**−0.0951**	.
	**0.1174**	0.0424	0.0941	0.0116	−0.0152	**0.1336**	**−0.0723**	.
**Diastolic blood** **pressure**	−0.0254	0.0698	−0.0033	0.0377	0.0625	−0.0146	0.0024	**0.7059**
	−0.0397	0.0576	−0.0106	0.0383	0.0860	−0.0370	−0.0067	**0.7199**
	−0.0338	0.0317	−0.0345	0.0318	0.0675	−0.0172	−0.0043	**0.7389**

For each pair of markers, the top, middle and bottom-lines show respectively partial correlation coefficients for 0, 1-19 and 20-148 unemployed quarters. Gray background indicates significant partial correlations with p<0.0001 while bold characters underline the strongest partial correlation coefficients r ≥ 0.1.

### Partial correlations between metabolic syndrome markers according to smoking status

Table 11 shows that the same pattern of partial correlations was observed whatever the smoking status. In participants who never smoked, former smokers or current smokers, there were strong correlations between systolic and diastolic blood pressures (r = 0.70, 0.71 and 0.71), between body mass index and waist (r = 0.53, 0.56 and 0.55) and hip (r = 0.64, 0.63 and 0.63) circumferences and between waist and hip circumferences (r = 0.10, 0.07 and 0.10); systolic blood pressure was correlated with waist (r = 0.15, 0.18 and 0.21) and hip (r = −0.09, −0.12 and −0.10) circumferences and with blood glucose (r = 0.14, 0.13 and 0.11), triglycerides with blood glucose (r = 0.10, 0.13 and 0.09), LDL (r = 0.29, 0.26 and 0.24), HDL (r = −0.23, −0.24 and −0.21) and waist circumference (r = 0.11, 0.11 and 0.12), HDL also with waist (r = −0.17, −0.17 and −0.14) and hip (r = 0.10, 0.10 and 0.09) circumferences, blood glucose with waist (r = 0.14, 0,17 and 0.15) and hip (r = −0.11, −0.12 and −0.10) circumferences.

**Table 11:** Partial correlations between metabolic syndrome markers according to smoking status.

	Blood glucose	Blood LDL cholesterol	Blood HDL cholesterol	Blood triglycerides	Body mass index	Waist circumference	Hip circumference	Systolic blood pressure
**Blood LDL** **cholesterol**	−0.0098	.	.	.	.	.	.	.
	−0.0634	.	.	.	.	.	.	.
	0.0105	.	.	.	.	.	.	.
**Blood HDL** **cholesterol**	0.0073	0.0578	.	.	.	.	.	.
	−0.0066	0.0155	.	.	.	.	.	.
	−0.0024	−0.0418	.	.	.	.	.	.
**Blood** **triglycerides**	**0.1039**	**0.2944**	**−0.2284**	.	.	.	.	.
	**0.1264**	**0.2576**	**−0.2404**	.	.	.	.	.
	**0.0903**	**0.2397**	**−0.2122**	.	.	.	.	.
**Body mass** **index**	0.0730	0.0551	−0.0563	0.0519	.	.	.	.
	0.0713	0.0371	−0.0734	0.0405	.	.	.	.
	0.0547	0.0618	−0.0782	0.0234	.	.	.	.
**Waist** **circumference**	**0.1428**	0.0394	**−0.1657**	**0.1097**	**0.5326**	.	.	.
	**0.1688**	0.0203	**−0.1682**	**0.1113**	**0.5632**	.	.	.
	**0.1518**	0.0633	**−0.1392**	**0.1217**	**0.5457**	.	.	.
**Hip** **circumference**	**−0.1061**	−0.0617	**0.0956**	−0.0517	**0.6376**	**0.0973**	.	.
	**−0.1196**	−0.0425	**0.1042**	−0.0542	**0.6278**	**0.0718**	.	.
	**−0.1044**	−0.0673	**0.0913**	−0.0614	**0.6344**	**0.0969**	.	.
**Systolic blood** **pressure**	**0.1409**	0.0647	0.0792	0.0127	−0.0084	**0.1498**	**−0.0882**	.
	**0.1337**	0.0383	0.0775	0.0098	−0.0042	**0.1790**	**−0.1207**	.
	**0.1082**	−0.0064	0.0278	0.0068	−0.0378	**0.2062**	**−0.0985**	.
**Diastolic blood** **pressure**	−0.0283	0.0511	−0.0153	0.0339	0.0713	−0.0206	−0.0009	**0.7046**
	−0.0395	0.0720	−0.0224	0.0321	0.0600	−0.0182	0.0064	**0.7129**
	−0.0049	0.0989	0.0463	0.0523	0.0725	−0.0255	−0.0034	**0.7080**

For each pair of markers, the top, middle and bottom-lines show respectively partial correlation coefficients for never, former and current smoking. Gray background indicates significant partial correlations with p<0.0001 while bold characters underline the strongest partial correlation coefficients r ≥ 0.1.

### Partial correlations between metabolic syndrome markers according to non-moderate alcohol consumption

The same pattern of partial correlations was observed across non-moderate alcohol consumption profiles (Table 12). In participants consuming rarely, sometimes or often non-moderate amounts of alcohol, there were strong correlations between systolic and diastolic blood pressures (r = 0.71, 0.71 and 0.71), between body mass index and waist (r = 0.55, 0.53 and 0.56) and hip (r = 0.67, 0.65 and 0.59) circumferences and between waist and hip circumferences (r = 0.08, 0.10 and 0.10); systolic blood pressure was correlated with waist (r = 0.14, 0.15 and 0.17) and hip (r = −0.08, −0.09 and −0.10) circumferences and with blood glucose (r = 0.13, 0.12 and 0.14), triglycerides with blood glucose (r = 0.14, 0.13 and 0.09), LDL (r = 0.28, 0.25 and 0.27), HDL (r = −0.23, −0.21 and −0.24) and waist circumference (r = 0.15, 0.12 and 0.10), HDL also with waist (r = −0.15, −0.18 and −0.17) and hip (r = 0.12, 0.11 and 0.10) circumferences, blood glucose with waist (r = 0.14, 0,15 and 0.16) and hip (r = −0.10, −0.11 and −0.12) circumferences.

**Table 12:** Partial correlations between metabolic syndrome markers according to non-moderate alcohol consumption.

	Blood glucose	Blood LDL cholesterol	Blood HDL cholesterol	Blood triglycerides	Body mass index	Waist circumference	Hip circumference	Systolic blood pressure
**Blood LDL** **cholesterol**	−0.0398	.	.	.	.	.	.	.
	−0.0239	.	.	.	.	.	.	.
	−0.0184	.	.	.	.	.	.	.
**Blood HDL** **cholesterol**	−0.0039	0.0513	.	.	.	.	.	.
	−0.0072	0.0332	.	.	.	.	.	.
	0.0058	0.0191	.	.	.	.	.	.
**Blood** **triglycerides**	**0.1422**	**0.2798**	**−0.2265**	.	.	.	.	.
	**0.1335**	**0.2487**	**−0.2127**	.	.	.	.	.
	**0.0882**	**0.2710**	**−0.2426**	.	.	.	.	.
**Body mass** **index**	0.0579	0.0478	−0.0691	0.0297	.	.	.	.
	0.0705	0.0556	−0.0596	0.0451	.	.	.	.
	0.0767	0.0550	−0.0543	0.0447	.	.	.	.
**Waist** **circumference**	**0.1447**	0.0176	**−0.1533**	**0.1491**	**0.5488**	.	.	.
	**0.1485**	0.0427	**−0.1808**	**0.1235**	**0.5265**	.	.	.
	**0.1624**	0.0391	**−0.1736**	**0.0998**	**0.5654**	.	.	.
**Hip** **circumference**	**−0.1002**	−0.0476	**0.1158**	−0.0706	**0.6692**	**0.0761**	.	.
	**−0.1077**	−0.0564	**0.1114**	−0.0669	**0.6490**	**0.0978**	.	.
	**−0.1178**	−0.0549	**0.0968**	−0.0513	**0.5953**	**0.0960**	.	.
**Systolic blood** **pressure**	**0.1275**	0.0576	0.0776	0.0087	−0.0150	**0.1442**	**−0.0845**	.
	**0.1247**	0.0491	0.0607	−0.0024	−0.0085	**0.1549**	**−0.0932**	.
	**0.1364**	0.0355	0.0658	0.0039	−0.0005	**0.1689**	**−0.1038**	.
**Diastolic blood** **pressure**	−0.0307	0.0526	−0.0292	0.0389	0.0787	−0.0327	0.0057	**0.7106**
	−0.0290	0.0587	−0.0021	0.0362	0.0725	−0.0177	−0.0011	**0.7137**
	−0.0247	0.0752	0.0048	0.0406	0.0581	−0.0138	−0.0004	**0.7075**

For each pair of markers, the top, middle and bottom-lines show respectively partial correlation coefficients for rarely, sometimes and often non-moderate alcohol consumption. Gray background indicates significant partial correlations with p<0.0001 while bold characters underline the strongest partial correlation coefficients r ≥ 0.1.

### Partial correlations between metabolic syndrome markers according to leisure-time physical inactivity

Table 13 shows that the same pattern of partial correlations was observed whatever the profile of leisure-time physical inactivity. In participants without or with leisure-time physical inactivity, there were strong correlations between systolic and diastolic blood pressures (r = 0.71 and 0.72), between body mass index and waist (r = 0.55 and 0.53) and hip (r = 0.63 and 0.66) circumferences and between waist and hip circumferences (r = 0.08 and 0.11); systolic blood pressure was correlated with waist (r = 0.17 and 0.18) and hip (r = −0.10 and −0.09) circumferences and with blood glucose (r = 0.13 and 0.13), triglycerides with blood glucose (r = 0.11 and 0.12), LDL (r = 0.27 and 0.25), HDL (r = −0.23 and −0.23) and waist circumference (r = 0.11 and 0.13), HDL also with waist (r = −0.16 and −0.16) and hip (r = 0.10 and 0.10) circumferences, blood glucose with waist (r = 0.16 and 0.16) and hip (r = −0.11 and −0.12) circumferences.

**Table 13:** Partial correlations between metabolic syndrome markers according to leisure-time physical inactivity.

	Blood glucose	Blood LDL cholesterol	Blood HDL cholesterol	Blood triglycerides	Body mass index	Waist circumference	Hip circumference	Systolic blood pressure
**Blood LDL** **cholesterol**	−0.0250	.	.	.	.	.	.	.
	−0.0428	.	.	.	.	.	.	.
**Blood HDL** **cholesterol**	0.0046	0.0319	.	.	.	.	.	.
	−0.0115	−0.0097	.	.	.	.	.	.
**Blood** **triglycerides**	**0.1096**	**0.2705**	**−0.2328**	.	.	.	.	.
	**0.1159**	**0.2481**	**−0.2291**	.	.	.	.	.
**Body mass** **index**	0.0702	0.0540	−0.0673	0.0405	.	.	.	.
	0.0666	0.0345	−0.0517	0.0299	.	.	.	.
**Waist** **circumference**	**0.1612**	0.0371	**−0.1589**	**0.1150**	**0.5487**	.	.	.
	**0.1575**	0.0544	**−0.1643**	**0.1263**	**0.5282**	.	.	.
**Hip** **circumference**	**−0.1136**	−0.0561	**0.0989**	−0.0560	**0.6315**	**0.0822**	.	.
	**−0.1174**	−0.0421	**0.1052**	−0.0763	**0.6560**	**0.1112**	.	.
**Systolic blood** **pressure**	**0.1339**	0.0433	0.0700	0.0064	−0.0097	**0.1707**	**−0.1016**	.
	**0.1259**	0.0171	0.0447	0.0130	−0.0175	**0.1775**	**−0.0866**	.
**Diastolic blood** **pressure**	−0.0278	0.0687	−0.0032	0.0361	0.0664	−0.0216	0.0032	**0.7102**
	−0.0224	0.0822	0.0216	0.0394	0.0693	−0.0035	−0.0177	**0.7193**

For each pair of markers, the top and bottom-lines show respectively partial correlation coefficients in participants without or with leisure-time physical inactivity. Gray background indicates significant partial correlations with p<0.0001 while bold characters underline the strongest partial correlation coefficients r ≥ 0.1.

### Partial correlations between metabolic syndrome markers according to diet quality

The same pattern of partial correlations was observed whatever diet quality (Table 14). In participants with no, slightly or strongly unbalanced diet, there were strong correlations between systolic and diastolic blood pressures (r = 0.72, 0.71 and 0.70), between body mass index and waist (r = 0.53, 0.55 and 0.56) and hip (r = 0.63, 0.64 and 0.64) circumferences and between waist and hip circumferences (r = 0.10, 0.08 and 0.08); systolic blood pressure was correlated with waist (r = 0.14, 0.17 and 0.19) and hip (r = −0.09, −0.10 and −0.09) circumferences and with blood glucose (r = 0.15, 0.13 and 0.13), triglycerides with blood glucose (r = 0.11, 0.11 and 0.13), LDL (r = 0.31, 0.26 and 0.26), HDL (r = −0.24, −0.23 and −0.23) and waist circumference (r = 0.13, 0.11 and 0.11), HDL also with waist (r = −0.16, −0.16 and −0.11) and hip (r = 0.10, 0.10 and 0.10) circumferences, blood glucose with waist (r = 0.16, 0,16 and 0.13) and hip (r = −0.21, −0.12 and −0.11) circumferences.

**Table 14:** Partial correlations between metabolic syndrome markers according to diet quality.

	Blood glucose	Blood LDL cholesterol	Blood HDL cholesterol	Blood triglycerides	Body mass index	Waist circumference	Hip circumference	Systolic blood pressure
**Blood LDL** **cholesterol**	−0.0219	.	.	.	.	.	.	.
	−0.0314	.	.	.	.	.	.	.
	−0.0245	.	.	.	.	.	.	.
**Blood HDL** **cholesterol**	0.0027	0.0619	.	.	.	.	.	.
	−0.0005	0.0243	.	.	.	.	.	.
	0.0121	−0.0057	.	.	.	.	.	.
**Blood** **triglycerides**	**0.1115**	**0.3107**	**−0.2441**	.	.	.	.	.
	**0.1085**	**0.2629**	**−0.2327**	.	.	.	.	.
	**0.1316**	**0.2589**	**−0.2304**	.	.	.	.	.
**Body mass** **index**	0.0667	0.0424	−0.0663	0.0431	.	.	.	.
	0.0690	0.0524	−0.0685	0.0414	.	.	.	.
	0.0770	0.0463	−0.0848	0.0255	.	.	.	.
**Waist** **circumference**	**0.1593**	0.0115	**−0.1590**	**0.1300**	**0.5303**	.	.	.
	**0.1653**	0.0368	**−0.1571**	**0.1152**	**0.5491**	.	.	.
	**0.1323**	0.0796	**−0.2070**	**0.1115**	**0.5649**	.	.	.
**Hip** **circumference**	**−0.1014**	−0.0328	**0.0964**	−0.0637	**0.6348**	**0.1014**	.	.
	**−0.1158**	−0.0549	**0.0989**	−0.0595	**0.6356**	**0.0812**	.	.
	**−0.1118**	−0.0719	**0.0957**	−0.0483	**0.6358**	**0.0828**	.	.
**Systolic blood** **pressure**	**0.1459**	0.0667	0.0865	0.0247	−0.0070	**0.1369**	**−0.0857**	.
	**0.1302**	0.0459	0.0739	0.0049	−0.0080	**0.1727**	**−0.1047**	.
	**0.1334**	−0.0004	0.0339	−0.0047	−0.0326	**0.1913**	**−0.0920**	.
**Diastolic blood** **pressure**	−0.0467	0.0413	−0.0208	0.0244	0.0592	−0.0110	0.0069	**0.7162**
	−0.0254	0.0669	−0.0041	0.0387	0.0639	−0.0182	0.0025	**0.7102**
	−0.0126	0.0140	0.0197	0.0424	0.0915	−0.0379	−0.0091	**0.6972**

For each pair of markers, the top, middle and bottom-lines show respectively partial correlation coefficients for no, slightly and strongly unbalanced diet. Gray background indicates significant partial correlations with p<0.0001 while bold characters underline the strongest partial correlation coefficients r ≥ 0.1.

## Discussion

The main finding of the present study is that the pattern of intercorrelations between major metabolic syndrome markers, analyzed as continuous variables, remains remarkably constant across a wide array of biological, social and behavioral characteristics of individuals. This pattern that notably includes intercorrelations of waist and hip circumferences with blood glucose, HDL, triglycerides and systolic blood pressure is observed whatever the sex, age, individual and parental histories of cardiovascular disease, social position, work environment, lifetime unemployment exposure, smoking status, non-moderate alcohol consumption, leisure-time physical inactivity and diet quality. It is even observed independently of the diagnosis of metabolic syndrome.

It is noteworthy that although all metabolic syndrome markers considered in this study are correlated with each other, partial correlation coefficients reveal that only some remain intercorrelated when correlations with the other markers are taken into account. Thus, the intercorrelations of diastolic blood pressure and body mass index with the other markers would in fact reflect their intercorrelations with systolic blood pressure and waist and hip circumferences. This supports the hypothesis that the effect of body mass index on cardiovascular risk would be almost entirely explained by waist and hip circumferences [28]. Furthermore, the strength of the intercorrelations of waist and hip circumferences with the other markers seems to indicate a prominent role of waist over hip circumference on cardiovascular risk as already suggested [29]. Likewise, even though diastolic and systolic blood pressures are highly correlated with each other, only the latter is correlated with the other markers, corroborating the view that it would have a prominent role over diastolic pressure in determining cardiovascular risk [30,31].

The mechanisms underlying the interrelationships between metabolic syndrome markers are not clear. One hypothesis is that genetic background would maintain, independently of individual’s characteristics, the coordinated expression of the genes that determine marker levels [32]. However, genome-wide association studies that looked for single nucleotide polymorphisms associated with marker levels have largely failed to identify the genetic dimension of metabolic syndrome as a whole [33]. The non-genetic nature of the stability of the interrelationships between metabolic syndrome markers would also be in accordance with the fact that neither individual nor parental histories of cardiovascular disease significantly modify the pattern of intercorrelations. A second hypothesis is that organs such as the intestine would regulate marker levels in a coordinated way. This would be corroborated by the existence of treatments targeting the intestine and having pleiotropic effects on marker levels [34,35]. This would also fit with the potential involvement of the gut microbiota in the development of metabolic syndrome [36]. Another organ playing such a role could be abdominal fat as reflected in body mass index whose level changes modulate several other markers, as glycemia and blood pressure [37,38], which largely mediate the excess risk of cardiovascular diseases associated with high body mass index [39].

Whatever the mechanisms underlying the interrelationships between metabolic syndrome markers, a remarkable feature besides their omnipresence is their relative insensitivity to individual’s characteristics that otherwise deeply affect marker levels. Thus, the same characteristics that have a strong influence on marker levels such as age [8,9], social position [10,11], work environment [12,13], smoking [14], heavy alcohol consumption [15], leisure-time physical inactivity [16] or unbalanced diet [17] seem to have little effect on the intercorrelations of markers with each other.

This study has some limitations. One is its external validity that is not guaranteed given that the findings were obtained in a cohort which was not representative of the French population, including in particular a large proportion of socially-privileged individuals. A second is that information on cardiovascular drug takings by participants was not available and could not be taken into account as confounding factors in the analyses.

In conclusion, the results indicate that the intercorrelations between several major metabolic syndrome markers are omnipresent in the general population, whatever individual’s characteristics. This observation provides a different angle of view on these markers whose relationships would extend well beyond the usual diagnosis of metabolic syndrome and that should be considered all together rather than separately in terms of etiology, prevention and treatment of metabolic diseases and cardiovascular risk.

## Supporting information

S1 Fig Lifetime unemployment exposure of participants. (DOCX)

S1 Table Indicators of social position of participants. (DOCX)

S2 Table Indicators of work environment of participants. (DOCX)

S3 Table Characteristics of cohort participants with or without missing values compared to randomly selected individuals from the French population. (DOCX)

S4 Table Means ± standard deviations of the distributions of metabolic syndrome markers in participants with or without missing values. (DOCX)
