# Supplementary figures and images for: Omnipresent intercorrelations of metabolic syndrome markers in the general population
Source: PLoS One. 2025 Aug 14;20(8):e0328577. doi: 10.1371/journal.pone.0328577 (PMC12352674; doi:10.1371/journal.pone.0328577)

**S1 Fig:** Lifetime unemployment exposure of participants.

**
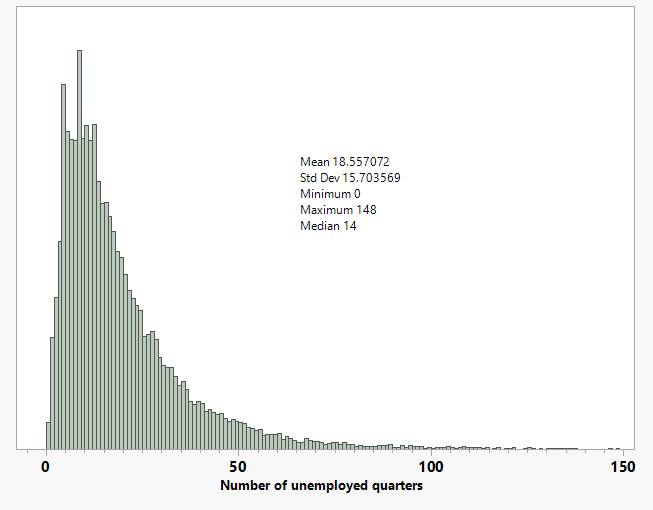
**

Supplement: S1 Fig — (DOCX) [file pone.0328577.s001.docx]
