# Supplementary material for: Omnipresent intercorrelations of metabolic syndrome markers in the general population
Source: PLoS One. 2025 Aug 14;20(8):e0328577. doi: 10.1371/journal.pone.0328577 (PMC12352674; doi:10.1371/journal.pone.0328577)
Supplement: S1 Table — (DOCX) [file pone.0328577.s002.docx]

# **S1 Table:** Indicators of social position of participants.

|  | | **n** | **%** |
| --- | --- | --- | --- |
| **Education (y)** | **≥17** | 40,704 | 25.5 |
|  | **14-16** | 56,200 | 35.2 |
|  | **12-13** | 25,669 | 16.1 |
|  | **≤11** | 35,212 | 22.1 |
|  | **Missing** | 1691 | 1.10 |
| **Occupation** | **Management** | 48,712 | 30.5 |
|  | **Intermediate** | 43,466 | 27.3 |
|  | **Blue collar/clerk** | 50,933 | 31.9 |
|  | **Missing** | 16,365 | 10.3 |
| **Income** | **Very high** | 46,493 | 29.2 |
|  | **High** | 48,467 | 30.4 |
|  | **Middle** | 39,631 | 24.9 |
|  | **Low** | 14,626 | 9.20 |
|  | **Missing** | 10,259 | 6.40 |
| **Spouse occupation** | **Management** | 37,978 | 23.8 |
|  | **Intermediate** | 29,849 | 18.7 |
|  | **Blue collar/clerk** | 43,526 | 27.3 |
|  | **Missing** | 48,123 | 30.2 |
| **Social vulnerability** | **Low** | 47,125 | 29.5 |
|  | **Average** | 64,238 | 40.3 |
|  | **High** | 47,454 | 29.8 |
|  | **Missing** | 659 | 0.4 |

# The percentages were calculated relatively to the number of participants selected for the study (n=159,476).
