# Supplementary material for: Omnipresent intercorrelations of metabolic syndrome markers in the general population
Source: PLoS One. 2025 Aug 14;20(8):e0328577. doi: 10.1371/journal.pone.0328577 (PMC12352674; doi:10.1371/journal.pone.0328577)
Supplement: S2 Table — (DOCX) [file pone.0328577.s003.docx]

**S2 Table:** Indicators of work environement of participants.

|  | | **n** | **%** |
| --- | --- | --- | --- |
| **Commuting time** | **<1h** | 86357 | 54.2 |
|  | **1h-2h** | 21876 | 13.7 |
|  | **>2h** | 4230 | 2.7 |
|  | **Missing** | 47013 | 29.5 |
| **Clocking in and out** | **No** | 87644 | 55.0 |
|  | **Yes** | 25423 | 15.9 |
|  | **Missing** | 46409 | 29.1 |
| **Regular working hours** | **No** | 62457 | 39.2 |
|  | **Yes** | 51135 | 32.1 |
|  | **Missing** | 45884 | 28.8 |
| **Long working hours** | **No** | 113252 | 71.0 |
|  | **Yes** | 46224 | 29.0 |
| **Night work** | **No** | 144628 | 90.7 |
|  | **Yes** | 14848 | 9.3 |
| **Dealing with the public** | **No** | 55840 | 35.0 |
|  | **Yes** | 22555 | 14.1 |
|  | **Missing** | 81081 | 50.8 |
| **Driving on public road** | **No** | 100151 | 62.8 |
|  | **Yes** | 13584 | 8.5 |
|  | **Missing** | 45741 | 28.7 |
| **Repetitive work** | **No** | 83361 | 52.3 |
|  | **Yes** | 27891 | 17.5 |
|  | **Missing** | 48224 | 30.2 |
| **Working with a screen** | **No** | 27996 | 17.6 |
|  | **Yes** | 85236 | 53.4 |
|  | **Missing** | 46244 | 29.0 |
| **Standing work posture** | **No** | 59193 | 37.1 |
|  | **Yes** | 54719 | 34.3 |
|  | **Missing** | 45564 | 28.6 |
| **Handling heavy loads** | **No** | 69358 | 43.5 |
|  | **Yes** | 44151 | 27.7 |
|  | **Missing** | 45967 | 28.8 |
| **Physically demanding work** | **No** | 113795 | 71.4 |
|  | **Yes** | 45681 | 28.6 |
| **Exposure to vibrations** | **No** | 108605 | 68.1 |
|  | **Yes** | 4153 | 2.6 |
|  | **Missing** | 46718 | 29.3 |
| **Exposure to noise** | **No** | 112172 | 70.3 |
|  | **Yes** | 47304 | 29.7 |
| **Outdoor work** | **No** | 103813 | 65.1 |
|  | **Yes** | 11684 | 7.3 |
|  | **Missing** | 43979 | 27.6 |
| **Working in the cold** | **No** | 110156 | 69.1 |
|  | **Yes** | 3887 | 2.4 |
|  | **Missing** | 45433 | 28.5 |
| **Working in the heat** | **No** | 108976 | 68.3 |
|  | **Yes** | 5313 | 3.3 |
|  | **Missing** | 45187 | 28.3 |
| **Exposure to chemicals** | **No** | 105297 | 66.0 |
|  | **Yes** | 54179 | 34.0 |
| **Effort-reward imbalance** | **Low** | 35447 | 22.2 |
|  | **Average** | 41700 | 26.1 |
|  | **High** | 34127 | 21.4 |
|  | **Missing** | 48202 | 30.2 |

# The percentages were calculated relatively to the number of participants selected for the study (n=159,476).
