# Supplementary material for: Omnipresent intercorrelations of metabolic syndrome markers in the general population
Source: PLoS One. 2025 Aug 14;20(8):e0328577. doi: 10.1371/journal.pone.0328577 (PMC12352674; doi:10.1371/journal.pone.0328577)
Supplement: S3 Table — (DOCX) [file pone.0328577.s004.docx]

**S3 Table:** Characteristics of cohort participants with or without missing values compared to randomly selected individuals from the French population.

|  | | **Representative**  **sample of the**  **French**  **population** | | **Whole**  **cohort** | | **Cohort**  **without**  **missing value** | | **Comparing**  **representative**  **sample of the**  **French**  **population**  **and whole**  **cohort** | **Comparing**  **representative**  **sample of the**  **French**  **population and**  **cohort without**  **missing value** | **Comparing**  **whole cohort**  **and cohort**  **without**  **missing value** |
| --- | --- | --- | --- | --- | --- | --- | --- | --- | --- | --- |
|  |  | **n** | **%** | **n** | **%** | **n** | **%** | **Cohen’s h** | **Cohen’s h** | **Cohen’s h** |
| **-** | **All** | 24,242 | - | 205,203 | - | 159,476 | - | - | - | - |
| **Sex** | **Women** | 12,745 | 52.6 | 110,193 | 53.7 | 84,266 | 52.8 | -0.022 | -0.004 | 0.018 |
|  | **Men** | 11,497 | 47.4 | 95,010 | 46.3 | 75,210 | 47.2 | 0.022 | 0.004 | -0.018 |
| **Age (y)** | **18-39** | 9657 | 39.9 | 66,832 | 32.6 | 50,782 | 31.8 | 0.152 | 0.169 | 0.017 |
|  | **40-54** | 7717 | 31.8 | 69,100 | 33.6 | 54,496 | 34.2 | -0.038 | -0.081 | -0.013 |
|  | **55-75** | 6868 | 28.4 | 69,271 | 33.8 | 54,198 | 34.0 | -0.117 | -0.121 | -0.004 |
| **Education** | **University** | 6022 | 24.9 | 118,646 | 58.9 | 96,904 | 61.4 | -0.705** | -0.744** | -0.039 |
|  | **Secondary school** | 11,643 | 48.0 | 33,246 | 16.5 | 25,669 | 16.3 | 0.694** | 0.705** | 0.011 |
|  | **Primary school** | 6577 | 27.1 | 49,538 | 24.6 | 35,212 | 22.3 | 0.057 | 0.116 | 0.059 |
| **Occupation** | **Management** | 3103 | 15.5 | 58,441 | 32.2 | 48,712 | 34.0 | -0.398* | -0.361* | 0.037 |
|  | **Intermediate** | 5060 | 25.2 | 54,114 | 29.9 | 43,466 | 30.4 | -0.105 | -0.048 | 0.058 |
|  | **Blue collar/clerk** | 11,900 | 59.3 | 68,817 | 37.9 | 50,933 | 35.6 | 0.432* | 0.558** | 0.126 |

The percentages were calculated relatively to the number of participants with or without missing values or of individuals randomly selected from the French population; Each pair of proportions was compared using Cohen’s h measure of effect size with the rule of thumb to categorize substantial differences as *small (0.2 ≤ h < 0.5), **medium (0.5 ≤ h > 0.8) or ***large (h ≥ 0.8).
