# Supplementary material for: Omnipresent intercorrelations of metabolic syndrome markers in the general population
Source: PLoS One. 2025 Aug 14;20(8):e0328577. doi: 10.1371/journal.pone.0328577 (PMC12352674; doi:10.1371/journal.pone.0328577)
Supplement: S4 Table — (DOCX) [file pone.0328577.s005.docx]

# **S4 Table:** Means ± standard deviations of the distributions of metabolic syndrome markers in participants with or without missing values.

|  | **Whole cohort** | | **Cohort without**  **missing value** | | **Cohen’s d** |
| --- | --- | --- | --- | --- | --- |
|  | **Mean** | **SD** | **Mean** | **SD** |  |
| **Blood glucose (mmol/l)** | 5.32 | 0.86 | 5.31 | 0.82 | 0.012 |
| **Blood HDL cholesterol (mmol/l)** | 1.51 | 0.41 | 1.51 | 0.40 | 0.001 |
| **Blood LDL cholesterol** **(mmol/l)** | 3.67 | 1.00 | 3.66 | 0.99 | 0.010 |
| **Blood triglycerides (mmol/l)** | 1.13 | 0.70 | 1.12 | 0.67 | 0.015 |
| **Body mass index (kg/m^2^)** | 25.0 | 4.5 | 24.9 | 4.4 | 0.022 |
| **Waist circumference (cm)** | 84.8 | 12.9 | 84.8 | 12.8 | 0.001 |
| **Hip circumference (cm)** | 99.3 | 8.7 | 99.2 | 8.5 | 0.005 |
| **Systolic blood pressure (mmHg)** | 128.1 | 16.1 | 128.0 | 15.8 | 0.006 |
| **Diastolic blood pressure (mmHg)** | 76.5 | 9.5 | 76.5 | 9.4 | 0.001 |

Differences between means were assessed using Cohen’s d measure of effect size with the rule of thumb to categorize substantial differences as *small (0.2 ≤ h < 0.5), **medium (0.5 ≤ h > 0.8) or ***large (h ≥ 0.8).
